# Subcellular Remodeling in Filamin C Deficient Mouse Hearts Impairs Myocyte Tension Development during Progression of Dilated Cardiomyopathy

**DOI:** 10.3390/ijms23020871

**Published:** 2022-01-14

**Authors:** Joseph D. Powers, Natalie J. Kirkland, Canzhao Liu, Swithin S. Razu, Xi Fang, Adam J. Engler, Ju Chen, Andrew D. McCulloch

**Affiliations:** 1 Department of Bioengineering, University of California San Diego, La Jolla, CA 92093, USA; nkirkland@eng.ucsd.edu (N.J.K.); srazu@eng.ucsd.edu (S.S.R.); aengler@eng.ucsd.edu (A.J.E.); juchen@health.ucsd.edu (J.C.); amcculloch@eng.ucsd.edu (A.D.M.); 2 Department of Medicine, University of California San Diego, La Jolla, CA 92093, USA; liucanzhao0521@163.com (C.L.); xifang@health.ucsd.edu (X.F.); 3 Institute for Engineering in Medicine, University of California San Diego, La Jolla, CA 92093, USA

**Keywords:** cardiac muscle, Z-disk, sarcomere, mechanotransmission, cellular remodeling, computational modeling

## Abstract

Dilated cardiomyopathy (DCM) is a life-threatening form of heart disease that is typically characterized by progressive thinning of the ventricular walls, chamber dilation, and systolic dysfunction. Multiple mutations in the gene encoding filamin C (FLNC), an actin-binding cytoskeletal protein in cardiomyocytes, have been found in patients with DCM. However, the mechanisms that lead to contractile impairment and DCM in patients with FLNC variants are poorly understood. To determine how FLNC regulates systolic force transmission and DCM remodeling, we used an inducible, cardiac-specific FLNC-knockout (icKO) model to produce a rapid onset of DCM in adult mice. Loss of FLNC reduced systolic force development in single cardiomyocytes and isolated papillary muscles but did not affect twitch kinetics or calcium transients. Electron and immunofluorescence microscopy showed significant defects in Z-disk alignment in icKO mice and altered myofilament lattice geometry. Moreover, a loss of FLNC induces a softening myocyte cortex and structural adaptations at the subcellular level that contribute to disrupted longitudinal force production during contraction. Spatially explicit computational models showed that these structural defects could be explained by a loss of inter-myofibril elastic coupling at the Z-disk. Our work identifies FLNC as a key regulator of the multiscale ultrastructure of cardiomyocytes and therefore plays an important role in maintaining systolic mechanotransmission pathways, the dysfunction of which may be key in driving progressive DCM.

## 1. Introduction

Dilated cardiomyopathy (DCM) is a common and life-threatening form of heart disease that affects 1 in ~250 individuals [1]. DCM is often characterized by systolic dysfunction, increased chamber volume, thin ventricular walls, and cardiomyocyte (CM) lengthening [1,2,3,4]. In non-ischemic DCM, patients often carry loss-of-function mutations in genes encoding sarcomeric or cytoskeletal proteins, many of which mediate mechanotransmission, mechanosensing, and/or mechanotransduction in cardiomyocytes. In particular, the costamere, which connects the myofilament lattice to the sarcolemma and extracellular matrix (ECM) via the Z-disks, contains many mechanosensitive proteins including integrins, vinculin, talin, and filamin C [5,6].

Filamin C (FLNC) is a striated muscle-specific isoform of the actin-crosslinking filamin family and associates with multiple proteins in the Z-disk and costamere, including integrins, desmin, and α-actinin [7,8]. FLNC is a large (~290 kDa) homo-dimeric protein that consists of 24 serially linked immunoglobulin-like domains that are interrupted by two flexible hinge regions, a C-terminal dimerization domain, and an N-terminal actin-binding domain that enables sarcomere binding at the Z-disk and facilitates thin filament anchoring [9]. To date, hundreds of unique FLNC variants have been linked to cardiomyopathies [10], including many in arrhythmogenic cardiomyopathy [11,12] and multiple FLNC-truncating mutations in patients with DCM [10,13,14,15]. Furthermore, a recent study [16] investigated 145 patients with FLNC-associated cardiomyopathies and found that sudden cardiac death occurred in about 19% of these patients, which was primarily associated with left-ventricular fibrosis. However, the role of FLNC in regulating cardiac mechanics and structure and the mechanisms by which the loss of full-length FLNC leads to a DCM phenotype are poorly understood.

To address this, we previously [17] created a genetically engineered murine model of inducible and cardiac-specific filamin C knockout (icKO) by crossing homozygous FLNC-floxed (FLNC^f/f^) mice with mice expressing the tamoxifen-inducible Cre-recombinase variant MerCreMer^+/−^ under the *myh6* promoter. This system enables mice to age into adulthood with normal cardiac function prior to inducing FLNC deletion via tamoxifen (Tam) injection. Control (Ctrl) mice are Tam-injected FLNC^f/f^ + MerCreMer^−/−^ littermates. By 2 weeks post-injection, FLNC protein expression is completely abolished in icKO mice ([17], see also Figure S1), and icKO hearts have significantly dilated ventricles and systolic deficiencies characteristic of a DCM phenotype [17]. However, the underlying mechanisms by which a loss of FLNC in adult mouse hearts leads to severe DCM remained unclear.

The aim of this study was to better understand the role of FLNC in the heart and elucidate mechanisms by which FLNC deletion in mouse hearts causes contractile impairment and progressive DCM remodeling. We employed the inducible, cardiac-specific icKO mouse model [17] in experimental and computational studies of cardiomyocyte structure-mechanics relations. In both the tissue and single-cell scales, loss of FLNC reduces systolic force development without affecting Ca^2+^ signaling or sarcomeric protein expression. Multiscale structural analyses revealed significantly disordered myofibrils and Z-disk orientation, including a slight compression and disorganization of the myofilament lattice in icKO cardiomyocytes compared with controls. We also found that FLNC deletion causes a reduction in the stiffness of the cardiomyocyte membrane/cortex. Finally, our multiscale, spatially explicit, computational models suggest that a primary mechanism of reduced contractility in icKO cardiomyocytes is a reduction of FLNC-dependent Z-disk rigidity that may inhibit transmission and homogeneity of sarcomere forces during systole. Our work presents a new integrated experimental and computational platform for future investigations into the relationship between cyto-architecture, Z-disk connectivity, and cardiac force development, and provides novel insights into mechanisms by which a loss of functional FLNC dysregulates this relationship in the development of DCM.

## 2. Results

### 2.1. FLNC Deletion Inhibits Contractility at the Cell and Tissue Level without Affecting Calcium Signaling

**Figure 1:**
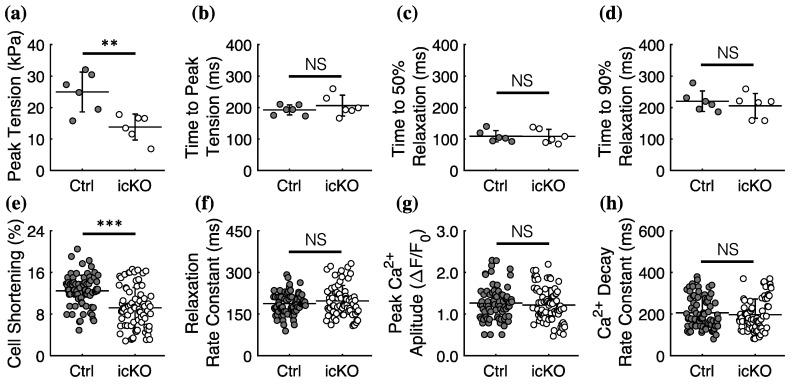
FLNC deletion in adult mouse hearts reduces cardiomyocyte contractility without affecting Ca^2+^ signaling. Peak twitch tension (**a**) in intact papillary muscles from icKO hearts was significantly reduced compared with controls. Time to peak twitch tension (**b**) and time to 50% (**c**) and 90% (**d**) relaxation (RT50 and RT90, respectively) were not different in papillary muscles from icKO vs. control hearts. Similar to the intact papillary muscles, intact single cells isolated from icKO hearts exhibit significantly reduced cell shortening (**e**) compared with cells from control hearts, while the relaxation rate constant (**f**) was not different between groups. The peak amplitude of the calcium transient (**g**) and cytosolic calcium decay rate constant (**h**) was not different between groups. ** *p* < 0.005, *** *p* < 0.0005, and NS = Not Significant for unpaired student’s *t*-test between groups. *N* = *n* = 6 for intact papillary muscles from each group, and *n* > 70 for isolated cells from each group from *N* = 6 animals in each group.

To determine the effects of FLNC deletion on cell and tissue-level systolic tension development, we measured the twitch mechanics and kinetics of electrically stimulated intact right-ventricular papillary muscles and single ventricular cardiomyocytes isolated from icKO and control hearts. Peak twitch tension in papillary muscles from icKO hearts was significantly reduced compared with controls (Figure 1a), while the twitch kinetics were not different between groups (Figure 1b,c). Similarly, the cell shortening (as a % of cell length) of stimulated single cardiomyocytes was significantly reduced in cells from icKO hearts compared with control hearts (Figure 1e), while the relaxation rate was not different between groups (Figure 1f). We simultaneously recorded Ca^2+^ transients during the twitch in single cardiomyocytes to determine whether FLNC deletion affects Ca^2+^ signaling properties in single cells. There were no differences in peak Ca^2+^ transient amplitude (Figure 1g) or Ca^2+^ decay rate (Figure 1h) between cardiomyocytes from icKO and control mice. Together, the results in Figure 1 show that FLNC deletion in adult mouse hearts reduced contractile force without affecting the calcium signaling in single cells.

### 2.2. FLNC Deletion in Adult Mouse Hearts Increases Z-Disk and Costamere Protein Expression, While Myofilament Protein Expression Is Largely Unaffected

Recent work [18] using human induced pluripotent stem cell-derived cardiomyocytes (hiPSC-CMs) has suggested that homozygous FLNC deletion causes significant reductions in thin filament protein expression, which inhibits the contractile forces of single hiPSC-CMs. We determined whether the functional effects of FLNC deletion on contractility and calcium signaling (Figure 1) are due to significant reductions in the expression of myofilament, Z-disk/Costamere, or Ca^2+^ signaling proteins. To do so, we analyzed our previously reported [17] transcriptomic and proteomic measurements from RNAseq and mass spectrometry of ventricular tissue samples from icKO and control hearts.

**Figure 2:**
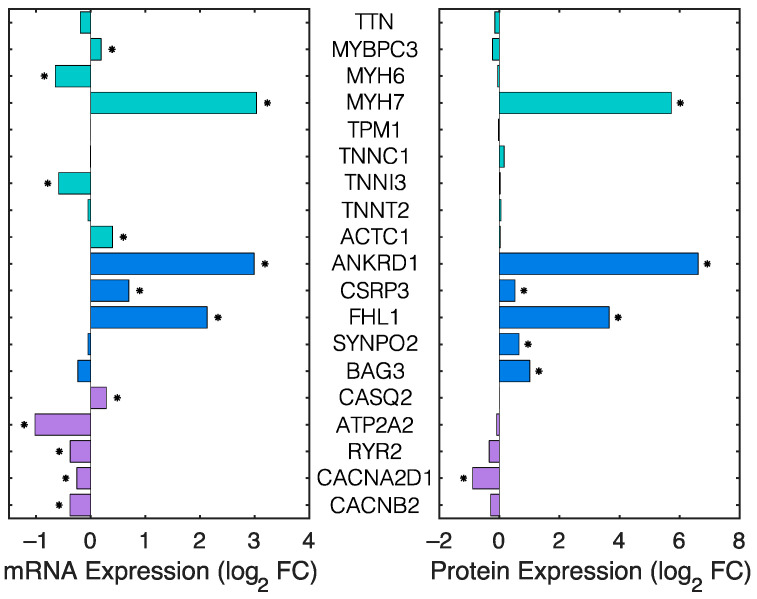
The effects of FLNC deletion in adult mouse hearts on mRNA and protein expression. The log_2_ fold-changes (FC) of mRNA expression (**left**) and protein expression (**right**) in icKO hearts relative to control hearts are shown for myofilament-associated proteins (teal), Z-disk/costamere-associated proteins (blue), and Ca^2+^ signaling-associated proteins (purple). Fold-changes in mRNA and protein expression were determined using RNA-seq and mass spectrometry (respectively) for *N* = 3 mice from each group, and * indicates *p* < 0.05. TTN: titin; MYBP3: myosin-binding protein C; MYH6: α-myosin heavy chain; MYH7: β-myosin heavy chain; TPM1: α-tropomyosin; TNNC1: cardiac troponin C; TNNI3, cardiac troponin I; TNNT2, cardiac troponin T; ACTC1, cardiac sarcomeric actin; ANKRD1: cardiac ankyrin repeat protein (CARP); CSRP3: muscle LIM protein; FHL1, four-and-a-half LIM domain protein 1; SYNPO2: synaptopodin-2; BAG3: co-chaperone Bcl2-associated athanogene 3; CASQ2: calsequestrin-2; ATP2A2, sarcoplasmic reticulum Ca^2+^-ATPase; RYR1, ryanodine receptor; CACNA2D1, voltage-dependent calcium channel subunit alpha-2/delta-1; CACNB2, calcium voltage-gated channel auxiliary subunit beta 2.

There was a statistically significant increase in the mRNA and protein expression of β-myosin (MYH7) in icKO hearts compared with controls, but there was no significant change in α-myosin (MYH6) protein expression (Figure 2) between icKO and control hearts. The increase in β-myosin expression had no effect on the twitch kinetics in icKO hearts compared with controls (Figure 1). Interestingly, expression levels of all other myofilament proteins investigated here were not different between groups (Figure 2, teal bars). Instead, the greatest changes in protein expression in icKO hearts were in proteins associated with the Z-disk/costamere (Figure 2, blue bars). We reported previously [17] that the protein abundances of vinculin, desmin, dystrophin, talin, β1D-integrin, focal adhesion kinase, and integrin-linked kinase are significantly increased in icKO hearts compared with controls, but there was not a significant change in α-actinin expression. Here, we report that there are also significant increases in the abundance of cardiac ankyrin repeat protein (CARP, or ANKRD1), muscle LIM protein (CRSP3), four-and-a-half LIM domain protein 1 (FHL1), synaptopodin-2 (SYNPO2), and the sarcomere-localized co-chaperone protein Bcl2-associated athanogene 3 (BAG3) in icKO hearts compared with controls (Figure 2). Moreover, the only significant change in expression of Ca^2+^ handling proteins that we saw was in the voltage-dependent calcium channel subunit alpha-2/delta-1 (CACNA2D1), which was significantly lower in icKO hearts than controls (Figure 2). However, this reduction in CACNA2D1 abundance in icKO hearts had no effect on the Ca^2+^ transient compared with controls (Figure 1g,h).

The results of our transcriptomic and proteomic analyses of icKO and control hearts (both from our previous report [17] and Figure 2) are two-fold: (i) the decreased contractility of icKO cardiomyocytes (Figure 1) cannot be explained by reductions in myofilament or Z-disk protein expression, as suggested in FLNC-null hiPSC-CMs [18], and (ii) deletion of FLNC in adult mouse hearts causes significant upregulation of many Z-disk/costamere proteins, which may be compensatory responses to the loss of FLNC in those compartments.

### 2.3. FLNC Deletion Causes Significant Cellular Growth and Subcellular Remodeling

**Figure 3:**
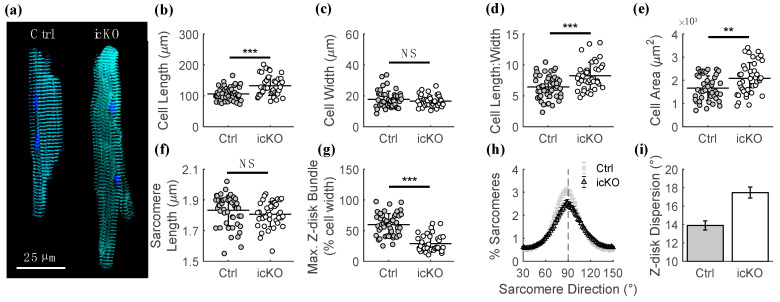
Adult cardiomyocytes undergo significant cellular structural remodeling in response to FLNC deletion. (**a**) Representative immunofluorescent images of control (left) and icKO (right) adult mouse ventricular cardiomyocytes, stained for the Z-disk protein α-actinin (cyan) and nuclei (DAPI, blue). The length of single cardiomyocytes (**b**) was significantly increased in cells from icKO hearts compared with controls, while the width (**c**) was not different. This leads to a significant increase in the length-to-width ratio (**d**) and surface area (**e**) of cardiomyocytes from icKO hearts compared with controls, indicative of DCM remodeling. Resting slack sarcomere length (**f**) was not different between genotypes, while maximum Z-disk transverse span ((**g**); as a % of cell width) was significantly reduced in cells from icKO hearts compared with controls. Fewer sarcomeres (**h**) had Z-disks aligned perpendicularly (90°) to the long axis of the cell in cardiomyocytes from icKO hearts (black) compared with control (gray), causing the angular dispersion of Z-disk orientation (**i**) to be greater in cardiomyocytes from icKO hearts compared with controls. ** *p* < 0.005, *** *p* < 0.0005, and NS = Not Significant for unpaired Student’s *t*-test.

Ruling out FLNC-dependent alterations in sarcomere protein content as a mechanism for reduced contractility in icKO hearts (Figure 1 and [17]), we hypothesized that FLNC deletion in cardiomyocytes induces subcellular structural remodeling that may disrupt normal ‘inside-out’ mechanotransmission (that is, the transmission of mechanical forces generated by the sarcomeres outward to the ECM and surrounding cells) [19]. To test this hypothesis, we used quantitative immunofluorescence microscopy (Figure 3a) to determine the effects of FLNC deletion on cellular morphology and subcellular structure.

Consistent with typical DCM remodeling in cardiomyocytes [20], FLNC deletion in adult mouse hearts caused significant cardiomyocyte lengthening (Figure 3b) without affecting cell width (Figure 3c). The length-to-width ratio and cell area of cardiomyocytes from icKO hearts were therefore significantly increased compared with cardiomyocytes from control mice (Figure 3d,e, respectively). While slack sarcomere length was not different in icKO cardiomyocytes compared with controls (Figure 3f), the maximum transverse Z-disk bundle length (as a % cell width) was significantly reduced in icKO cardiomyocytes compared with controls (Figure 3g), suggesting that a loss of FLNC causes a disruption in inter-myofibril Z-disk registration. Moreover, the percent of Z-disks that are perpendicular (90°) to the long-axis of the cardiomyocyte is reduced in icKO cardiomyocytes compared with controls (Figure 3h). That is, there is greater angular dispersion of Z-disk orientation in icKO cardiomyocytes compared with controls (Figure 3i). Together, the results shown in Figure 3 demonstrate that FLNC deletion in adult mouse hearts induces eccentric cardiomyocyte growth and a significant reduction in Z-disk alignment.

**Figure 4:**
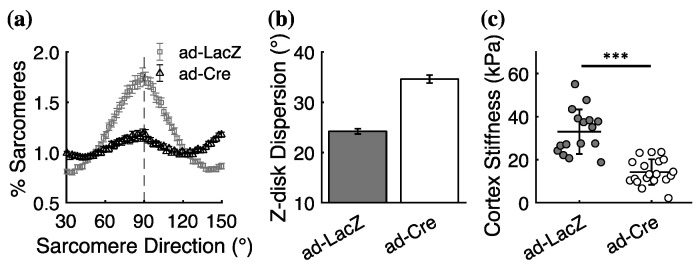
Neonatal ventricular mouse cardiomyocytes (NMVCMs) also exhibit significant structural and mechanical adaptations in response to FLNC deletion. (**a**) Fewer sarcomeres have Z-disks aligned perpendicularly (90°) to the long axis of the cell in NMVCMs infected with ad-Cre (FLNC-knockdown; black symbols) compared with ad-LacZ (controls; gray symbols), causing the angular dispersion of Z-disk orientation (**b**) to be greater in ad-Cre NVMCMs with ad-LacZ. (**c**) AFM revealed a significant decrease in the passive transverse stiffness of the cell membrane/cortex of NMVMs lacking FLNC (ad-Cre) compared with controls (ad-LacZ). *** *p* < 0.0005 for unpaired student’s *t*-test. *N* = 2 and *n* = 16 for ad-LacZ, *N* = 2 and *n* = 19 for ad-Cre.

To determine whether FLNC deletion similarly affects cellular structure and mechanics of neonatal mouse ventricular cardiomyocytes (NMVCMs), we infected FLNC^f/f^ NMVCMs with an adenovirus that expresses either β-galactosidase (ad-LacZ) as a control or Cre-recombinase (ad-Cre) to knock out FLNC in vitro. We quantified the sarcomeric organization of control and FLNC-knockdown NMVCMs that had been cultured for 96 h on nanopatterned topography to promote sarcomeric alignment (see Materials and Methods). We also used atomic force microscopy (AFM) to determine the effects of FLNC deletion on the transverse stiffness of the cellular membrane/cortical cytoskeleton. Similar to adult cardiomyocytes, FLNC deletion in NMVCMs causes a significant reduction in the number of sarcomeres aligned perpendicularly with the long axis of the cell (Figure 4a) and an increase in the angular dispersion of Z-disks (Figure 4b). Interestingly, AFM revealed that the transverse indentation stiffness indicative of cortical tension in NMVCMs lacking FLNC was significantly lower than in controls (Figure 4c). These results suggest that the subcellular structural remodeling due to FLNC deletion in cardiomyocytes may be associated with decreased cortical tension.

### 2.4. Spatially Explicit Computational Models Informed by Subcellular Structural Measurements Describe a Potential Role of FLNC in Mediating Z-Disk Inter-Connectivity

**Figure 5:**
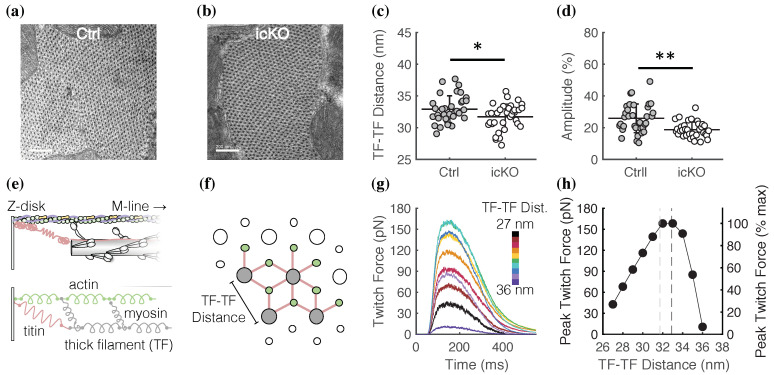
Myofilament lattice geometry determined by quantitative analysis of TEM images of papillary muscle cross-sections informs a spatially explicit computational model of a cardiac half-sarcomere. Example TEM images of a papillary muscle cross-section from a control (**a**) and an icKO (**b**) heart. Scale bar is 200 nm. (**c**) Average thick filament (TF) center-to-center separation distance (**c**) was significantly reduced in icKO cardiomyocytes compared to controls. The amplitude of the Gaussian fit to the histogram of distributions of TF-TF distances ((**d**); as a % of total TFs measured per image) was significantly lower in icKO cardiomyocytes than controls, suggesting less uniformity of TF separation distance in icKO cardiomyocytes than in controls. * *p* < 0.05, ** *p* < 0.005 using an unpaired Student’s *t*-test. *n* > 30 images analyzed from *N* = 2 samples for each group. Each image contained >200 TFs. (**e**) Schematic of our previously described [25,26] computational model, which consists of 4 thick filaments (TFs, gray), 8 actin (thin) filaments (green), and 14 titin filaments (pink) with periodic boundary conditions to simulate a semi-infinite lattice (indicated by the white filaments). (**f**) A cross-section showing the 3D arrangement of the myofilament lattice (color scheme is the same as panel (**e**)). (**g**) Cardiac twitch force transients for a range of TF-TF separation distances (27–36 nm) for a constant sarcomere length (1.85 µm). Each trace is an average of 50 twitch simulations. (**h**) The peak twitch force for each TF-TF distance from panel (**g**). The black and gray vertical dashed lines indicate the experimentally determined TF-TF distances for control and icKO cardiomyocytes, respectively.

Our previous work [21] in vinculin-null mice revealed a vinculin-regulated relationship between the cortical stiffness of cardiomyocytes and the myofilament lattice spacing, which, in turn, affects sarcomere force production. As such, we hypothesized that the cortical softening of FLNC-null cardiomyocytes (Figure 4c) also affects myofilament lattice geometry. To test this, we quantified myofilament lattice geometry by using transmission electron microscopy (TEM) to image 60-nm-thick cross-sections of cardiac papillary muscles from control (Figure 5a) and icKO (Figure 5b) hearts. We measured the center-to-center distance between every thick filament (TF) in the image and fit the resulting histogram of the distribution of distances with a Gaussian to determine the average TF-TF distance. We used the amplitude of the Gaussian fit as a proxy for the consistency in the separation distance between TFs (similar to the intensity of a 1,0 reflection of an *X*-ray diffraction pattern [22]). We found that the average TF-TF distance was significantly decreased in icKO cardiomyocytes compared with controls (Figure 5c). Moreover, the amplitude of the histogram of the distribution of distances is significantly reduced in icKO cardiomyocytes compared with controls (Figure 5d). These results demonstrate that the subcellular remodeling and reduced cortical tension induced by FLNC deletion in cardiomyocytes include a moderate but significant compression and disorganization of the myofilament lattice.

Next, to test whether the 1.2-nm compression of the myofilament lattice in icKO cardiomyocytes (Figure 5c) can account for the reduction in twitch force (Figure 1), we employed our spatially explicit computational model of a half-sarcomere [23,24,25,26], which was updated to simulate cardiac muscle twitches (see Materials and Methods). The model consists of thick filaments, thin filaments, and titin (Figure 5e) arranged in a 3D lattice with double-hexagonal symmetry (Figure 5f). We set the sarcomere length constant at 1.85 µm based on our measurements (Figure 3f), varied the TF-TF separation distance across a range that spans our measured TF-TF distances (Figure 4), and simulated twitch force transients for each case (Figure 5g). Our model predicts that a change in TF-TF separation distance from 27 nm to 36 nm significantly affects the peak twitch force, with a maximum twitch force occurring between 32 and 33 nm (Figure 5h). Interestingly, we found that the myofilament lattice compression in icKO cardiomyocytes was not predicted to account for the reduction in peak twitch force measured in these cells (Figure 1). As indicated by the vertical dashed lines in Figure 5h, the reduction in TF-TF separation distance from 32.9 nm in control cardiomyocytes (black dashed line) to 31.7 nm in icKO cardiomyocytes (gray dashed line) was predicted to decrease peak twitch force by ~5%.

**Figure 6:**
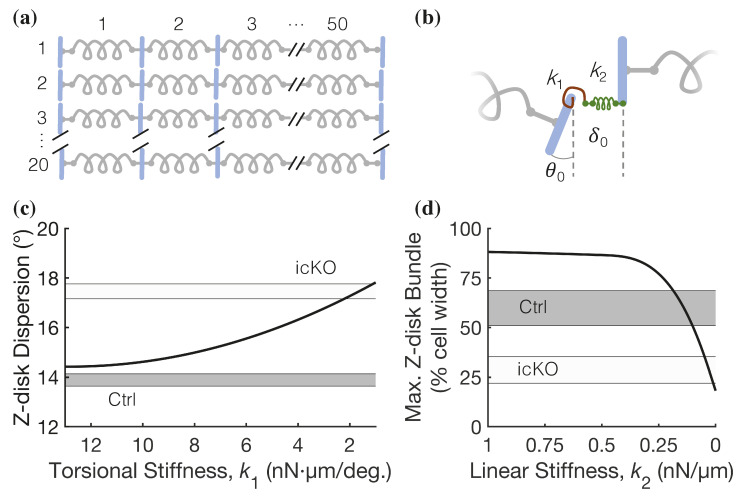
Cell-level model of the effects of spatial organization and inter-myofibril connectivity of sarcomeres on cell mechanics. (**a**) Schematic of our new spatially explicit computational model of a cardiomyocyte with 1000 sarcomeres (gray springs). (**b**) Adjacent Z-disks (blue) are mechanically coupled by a torsional (red) and a linear (green) spring system with stiffness *k*_1_ and *k*_2_, respectively. (**c**) The model predicts that the stiffness of the torsional spring (*k*_1_) regulating the orientation of the Z-disk must decrease by ~1 order of magnitude to account for the increase in Z-disk angular dispersion (Figure 3h) in icKO cardiomyocytes compared with control cardiomyocytes (gray and black dashed lines, respectively). (**d**) When decreasing the stiffness of the linear spring (*k*_2_) and regulating the distance (δ_0_) between adjacent Z-disks from 1–0 nN/µm, the model predicts that the maximum Z-disk bundle (as a % of cell width) decreases from above the value measured for control cardiomyocytes (black dashed line) to below that of icKO cardiomyocytes (gray dashed line).

Because our computational model of the half-sarcomere predicted that the compression of the myofilament lattice in icKO cardiomyocytes contributes only partially to the observed loss of twitch tension, we hypothesized that contractile dysfunction in icKO cardiomyocytes is dependent on myocyte ultrastructural remodeling (Figure 3). To test this, we developed a new spatially explicit computational model of myofibrillar organization that simulates the effects of altered connectivity between myofilaments at the Z-disk on Z-disk orientation and separation in the cardiomyocyte (Figure 6). The model consisted of 1000 sarcomeres (50 in series and 20 in parallel; Figure 6a) in which Z-disks were coupled by a torsional spring and a linear spring system (Figure 6b). The distribution of sarcomere lengths was prescribed based on our measurements in single cardiomyocytes (Figure 3e).

To test the hypothesis that FLNC is involved in maintaining the connectivity and orientation of adjacent Z-disks across myofibrils, we used our cell-level model to independently investigate the sensitivity of the angular dispersion of Z-disks on the stiffness of the torsional spring (*k*_1_; Figure 6b) and the Z-disk alignment on the lateral elastic coupling of adjacent myofibrils. The dispersion of Z-disk angles (θ_0_; Figure 6b) was measured relative to a vertical orientation (θ_0_ = 0°), and Z-disks were considered ‘bundled’ if the absolute value of their offset distance (δ_0_; Figure 6b) was ≤50 nm (i.e., half the width of the Z-disk). Keeping all other parameters constant (and *k*_2_ = 35 nN/µm), the model predicted that decreasing *k*_1_ by approximately one order of magnitude increased the average angular dispersion of Z-disks from approximately that measured in control cardiomyocytes (13.9 ± 0.5°, dark shaded region in Figure 6c) to beyond that in icKO cardiomyocytes (17.5 ± 0.5°, light shaded region in Figure 6c). Thus, by decreasing only *k*_1_, the model predicts an increase in Z-disk angular dispersion similar to that observed in icKO cardiomyocytes. Moreover, when *k*_2_ = 1 nN/µm, the maximum Z-disk bundle is predicted to be 90% of the cell width (Figure 6d), which is greater than what we measured in control cardiomyocytes (60 ± 9%, dark shaded region). However, decreasing *k*_2_ to 0 nN/µm, the maximum Z-disk bundle is predicted to progressively decrease to only 20% of the cell width (Figure 6d), which is less than what we measured in icKO cardiomyocytes (28 ± 7%, gray dashed line). Thus, by decreasing only *k*_2_, the model predicts a decrease in Z-disk bundling similar to (and beyond) that of icKO cardiomyocytes. Together, these results suggest that FLNC could play an important role in maintaining sarcomere organization and alignment in adult mouse cardiomyocytes by stiffening the trans-myofibril elastic coupling of Z-disks.

## 3. Discussion

### 3.1. The Role of Filamin C in Regulating Subcellular Ultrastructure to Mediate ‘Inside-Out’ Mechano-Transmission of Systolic Forces

The goal of this study was to elucidate the role of filamin C (FLNC) in regulating cardiac systolic contractile function and DCM [17] by measuring the structural and mechanical alterations in mouse cardiac muscle lacking FLNC and using multiscale models to investigate the mechanisms of altered force transmission and Z-disk coupling. To do so, we measured myocyte mechanics and structure in genetically engineered mice that enable inducible and cardiac-specific deletion of FLNC [17] and used computational models to investigate multiscale structure-function mechanisms. Rather than a loss of sarcomere protein expression [18] or altered calcium signaling, we conclude that the primary mechanism of reduced contractility in icKO adult mouse cardiomyocytes may involve ultrastructural rearrangement of myofibrils and dysregulated Z-disk orientation that can impair lattice spacing and the development and transmission of sarcomeric forces. This remodeling may be due to a combination of weakened inter-myofibril connectivity and softened cell cortex upon FLNC deletion. Thus, our work suggests a role of FLNC in regulating cell mechanics, Z-disk alignment and orientation, and inter-myofibril connectivity in the mammalian heart, which putatively mediate proper intracellular transmission of sarcomere forces.

The inducible and cardiac-specific FLNC knockout (icKO) mouse model represents a useful tool for investigating the role of FLNC in cardiac structure and systolic force transmission in adult cardiomyocytes while circumventing the embryonic lethality of global FLNC deletion in mice [17,27]. Other researchers have recently used gene-edited hiPSC-CMs to investigate the role of FLNC deletion and FLNC variants in cardiac contractility [18]. Interestingly, the authors report that homozygous deletion of FLNC in hiPSC-CMs results in dysregulated sarcomere assembly, organization, and sarcomere protein expression [18]. In agreement with their study [18], we here report significant sarcomere disorganization in icKO cardiomyocytes compared with controls. However, we do not observe a significant loss of sarcomere content or protein expression (Figure 2 and Figure 3a). This discrepancy may arise from differences in the biological model used in each study. Namely, because our inducible mouse model enables us to delete FLNC in adult cardiomyocytes that have fully matured in vivo, we are able to investigate the role of FLNC in maintaining (rather than assembling) sarcomeres and myofibrils in the heart. However, in an in-vitro hiPSC-CM model, homozygous FLNC-knockout hiPSC-CMs are differentiated and matured without FLNC [18], potentially complicating the discernment between the role of FLNC in regulating myofibril mechanical integrity in vivo versus its role in cardiomyocyte differentiation, sarcomerogenesis, and maturation processes in vitro.

Our findings are in good agreement with a recent study [28] in which mutated FLNC in medaka was found to cause a breakdown of myofibril stability in both cardiac and skeletal muscle. In the skeletal muscle of the FLNC-variant fish, inter-myofibril connections appeared to be somewhat impaired. Inhibiting contractility alleviated some of the muscle damage, suggesting that FLNC may play a critical role in maintaining the structural integrity of myofibrils and mediating the transmission of sarcomere tension through the myofilament lattice and myofibril network. Our subcellular computational model also agrees well with a similar model of skeletal muscle [29] in which the role of inter-myofibrillar connections in regulating isometric force was investigated. That model predicted that isometric force is reduced when the inter-myofibrillar connection is lost, consistent with experimental findings using skeletal muscle from desmin-null mice [30]. Furthermore, when the stiffness of the connection between myofibrils is zero, sarcomere length is more heterogeneous after contraction compared to stiffer inter-myofibrillar connections. Thus, our findings together with these previous studies [29,30] suggest that FLNC may play a similar role as intermediate filaments in maintaining the sarcomere registration and myofibril architecture required for optimal systolic force production.

### 3.2. Limitations and Future Directions

There are limitations in the present study. A significant limitation in using electron micrographs to quantify myofilament lattice geometry is that the isolation, fixation, and sectioning of the cardiac muscle preparations (see Materials and Methods) almost certainly affects the subcellular structure and organization of the myofilaments compared to the in vivo environment. Therefore, it is difficult to quantitatively compare the TF-TF distance measurements we report here (Figure 4c) with lattice spacing measurements of intact rodent cardiac muscle [31,32,33,34]. Future work using *X*-ray diffraction techniques is needed to more rigorously quantify the effects of FLNC deletion on the structural dynamics of the myofilaments in cardiac muscle in situ. Nonetheless, our thick filament spacing measurements (Figure 4) are in very good quantitative agreement with analogous measurements previously reported [21] using similar electron microscopy approaches with resting murine cardiac muscle. Secondly, an expansion of our cell-level computational model (Figure 6) is needed to simulate the effects of disordered myofibril alignment and weakened Z-disk connections on contractile mechanics, as well as the interaction sensitivity of both a decreased torsional and linear Z-disk attachment stiffness. Future work will be aimed at integrating the half-sarcomere (Figure 5) and cell-level model (Figure 6) to develop a single multiscale mechanical model of the cardiomyocyte, informed by experimentally determined structural information, that can predict contractile deficits caused by DCM-causing protein variants or deletions.

## 4. Materials and Methods

### 4.1. Animal Use & Ethics

All animal experiments were done in accordance with protocols approved by the University of California San Diego Institutional Animal Care and Use Committee and followed The Guide for the Care and Use of Laboratory Animals [35]. Adult (10 weeks of age) male and female mice were euthanized following the procedures approved by the Institutional Animal Care and Use Committee for the University of California San Diego.

### 4.2. Inducible and Cardiac-Specific FLNC-Knockout (icKO) Mouse Model

The animal model used in this study was described previously [17]. Briefly, inducible FLNC deletion in adult mouse hearts was achieved using a tamoxifen-induced Cre-Lox system that completely deleted FLNC protein expression by 2 weeks-post induction. Adult (8 weeks of age) male and female littermates were injected with tamoxifen (dissolved in peanut oil) at 40 mg/kg/day over 3 days via intraperitoneal injection. Control mice used in all experiments were FLNC^f/f^ + MerCreMer^−/−^ + Tamoxifen and icKO mice were FLNC^f/f^ + MerCreMer^+/−^ + Tamoxifen, as we have done previously [17]. Mice were used exactly 2 weeks post-injection for all experiments.

Mice were sedated by inhalation of isoflurane and an intraperitoneal (IP) injection of 0.1 mL of heparin was administered to minimize blood clotting in the ventricles. Approximately four minutes after the injection of heparin, cervical dislocation was performed to euthanize the animal, followed by rapid excision of the heart.

### 4.3. Intact Papillary Muscle Mechanics

Intact cardiac muscle mechanics were performed as described previously [36]. Briefly, hearts were rapidly excised via thoracotomy, and immediately immersed in oxygenated (95% O_2_, 5% CO_2_), room temperature Krebs buffer containing (in mM) 118.5 NaCl, 5 KCl, 1.2 MgSO_4_, 2 NaH_2_PO_4_, 25 NaHCO_3_, 1.8 CaCl_2_, and 10 glucose. Hearts were then rinsed via aortic retrograde perfusion with Krebs buffer containing low calcium (0.1 mM CaCl_2_) with 20 mM 2,3-Butanedione 2-monoxime (BDM) to minimize contraction and subsequent damage during dissection.

Small intact papillary were carefully dissected from the right ventricle and mounted between a force transducer (Aurora Scientific, Aurora, Ontario, Canada, Model 400A) and a length-controlling motor (Aurora Scientific, Aurora, Ontario, Canada, Model 300C). Each end of the papillary was attached to custom arms of the motor and force transducer made from insect pins. The papillary muscle was then submerged in an experimental chamber (Aurora Scientific, Aurora, Ontario, Canada, Model 1500A) that was continuously perfused with modified Krebs buffer (1.8 mM CaCl_2_) at room temperature. Twitches were elicited by field stimulation with platinum plate electrodes at 1 Hz with oscillating polarity. Papillary muscles were initially mounted at slack length and paced for 20 min prior to data collection to ensure no damage had been done to the muscle during dissection. Papillary muscles with twitch tension runoff of >15% were assumed damaged and the sample was discarded. Papillary muscles were then slowly stretched by 15% of their initial length to reach a plateau in maximum peak twitch tension with minimal passive tension development (assumed to be SL ~2.3 µm). Continuous twitch tension traces were recorded using custom LabView software at a sampling rate of 1 kHz and were analyzed with custom code written using MATLAB software (version 2018a, The MathWorks, Natick, MA, USA).

### 4.4. Intact Single-Cell Shortening and Calcium Imaging

Adult male and female mouse cardiomyocytes were isolated with a Langendorff system, as previously described [37,38]. Cardiomyocyte calcium transients and cell shortening/re-lengthening measurements were made as previously described [39,40]. Briefly, the cells were loaded with Fura-2-AM (1.0 μmol/L, 20 min) and electrically stimulated at a frequency of 0.5 Hz. Cell shortening was assessed using a video-based edge-detection system (IonOptix, Milton, MA, USA), while the amplitude of intracellular Ca^2+^ transient was simultaneously determined by the change between the basal and peak Fura-2-AM ratio (Δ*F*/*F*_0_). The decay of the Ca^2+^ transient (time to 63% decline) was used to determine the rate constant (Tau) of cytosolic calcium decay. The amplitude of cell contraction was assessed by peak shortening and the rate of cell relaxation rate constant was assessed by the time to 63% re-lengthening (Tau). A total of 60–80 individual cardiomyocytes from 4 mice of each group were recorded and analyzed.

### 4.5. Ventricular Cardiomyocyte Isolation and Quantitative Immunofluorescence

Hearts were rapidly excised via thoracotomy and immediately immersed and rinsed via aortic retrograde perfusion in room temperature Perfusion Buffer (PB; pH 7.35) containing (in mM) 113 NaCl, 4.7 KCl, 0.6 KH_2_PO_4_, 0.6 Na_2_HPO_4_, 0.032 phenol red, 1.2 MgSO_4_·7H_2_O, 12 NaHCO_3_, 10 KHCO_3_, 10 HEPES, 30 Taurine, and 5.5 glucose. Hearts were then suspended from a Langendorff perfusion system and perfused with PB at 37 °C for 5 min at a flow rate of 2 mL/min. Hearts were then perfused with PB containing 500 U/mg collagenase and 12.5 µM CaCl_2_ at 2 mL/min until hearts were visibly digested (~10–30 min), at which point the atria were discarded and the ventricles were minced in PB with 12.5 µM CaCl_2_ and 10% Fetal Bovine Serum (FBS) at 37 °C for ~5 min. Cells were then gently isolated by gently triturating the minced tissue in 37 °C PB with 5% FBS and 12.5 µM CaCl_2_ and passed through a 100 µm cell strainer.

Isolated ventricular cardiomyocytes were cultured on a laminin-coated (2 µg/cm^2^) glass coverslip and incubated at 37 °C and 2% CO_2_ for 3 h in Hanks’ Balanced Salt solution (HBSS, gibco) containing 2% MEM Amino Acids, 1% MEM Vitamins, 1% L-glutamine, 0.4% Penicillin/Strep 0.5 mM CaCl_2_, 0.8 mM MgSO_4_, and 0.6 mM KCl (pH 7.2). Cells were then rinsed in room-temperature Dulbecco’s Phosphate Buffered Saline (DPBS, gibco) and fixed by incubation in 2% paraformaldehyde (in DPBS) for 10 min at room temperature with gentle rocking. Cells were then rinsed 3 times in room temperature DPBS (5 min each with gentle rocking) prior to being permeabilized by incubation in room temperature 0.25% triton (in DPBS) solution for 5 min with gentle rocking. Cells were ‘blocked’ by incubation in DPBS containing 5% goat serum and 10% bovine serum albumin (BSA) for 1 h at room temperature with gentle rocking. Cells were then co-stained with primary antibodies (see Table S1) in DPBS with 10% BSA for 2 h at room temperature with gentle rocking. Next, cells were co-stained with secondary antibodies (see Table S2) in DPBS with 10% BSA for 30 min at room temperature with gentle rocking in the dark. Cells were then rinsed in DPBS and treated with DAPI (nuclear stain) plus ProLong Gold Antifade mountant (ThermoFisher, P36931).

Cells were imaged with an Evos2 automated microscope at 40× magnification using an oil-immersion objective lens. Images were analyzed using ImageJ and custom code written in MATLAB software (version 2018a). Similar to previous work [41], the angular dispersion of Z-disks was calculated using the images of α-actinin and the local gradient orientation plugin in ImageJ. Sarcomere lengths were determined by measuring the distance between Z-disks for a large region of interest (≥30 sarcomeres) in each cardiomyocyte. The maximum Z-disk bundle width was measured using ImageJ software, as others have done previously [42].

### 4.6. Transmission Electron Microscopy (TEM) of Cardiomyocyte Cross-Sections and Image Analysis

Adult male and female littermate mouse hearts with and without FLNC (see Section 4.2) were rapidly excised via thoracotomy and immediately immersed and perfused (via retrograde aortic perfusion) with oxygenated (95% O_2_, 5% CO_2_), room temperature Tyrode solution (pH 7.4) containing (in mM), 137 NaCl, 4 KCl, 0.5 MgCl_2_, 10 HEPES, and 5 glucose. Hearts were then perfused with a fixative solution containing 2% paraformaldehyde and 2.5% glutaraldehyde in 0.15 M sodium cacodylate (SC) buffer (pH 7.4). After fixation, the left ventricle was carefully opened, and left ventricular papillary muscles were dissected. Papillary muscles were then treated with 1% osmium in 0.15 M SC buffer for 1–2 h on ice (4 °C), after which they were washed 5 times (10 min each) in 0.15 M SC buffer followed by rinsing in ddH_2_O on ice. Papillary muscles were then incubated in 2% UA for 1–2 h at 4 °C. Next, papillary muscles were dehydrated by sequentially incubating them in progressively increased concentrations of EtOH (50%, 75%, 90%, and 2 × 100%) for 10 min each at 4 °C. They were then incubated in dry acetone for 15 min at room temperature, followed by a 1-h incubation in 50:50 EtOH:Durcupan at room temperature. Finally, papillary muscles were incubated in 100% Durcupan overnight.

The following day, papillary muscles were rinsed in fresh 100% Durcupan for ~4 h at room temperature. Tissues were then embedded in Durcupan at 60 °C for 36–48 h. Ultrathin sections (60 nm) were cut perpendicularly to the long axis of the papillary muscle using a Leica microtome with a diamond knife and were then stained with uranyl acetate and lead. Images of the papillary cross-sections were captured on a JEOL 1400 plus transmission electron microscope (TEM) at 80 kV with a Gatan 4 k × 4 k camera and a magnification of 40,000× and 4096 × 4096 pixels.

TEM images were analyzed using ImageJ software and custom MATLAB code. Images of the cross-section of the myofilament lattice (see Figure 4) were first blurred with a Gaussian filter (radius 10 pixels) in ImageJ and then made binary. Using the object counter plugin in ImageJ (filter size of 1000–5000 pixels^2^, threshold 128) on the binary image, individual thick filaments (TFs) were mapped onto a 2D grid and the center-to-center distance between each TF (>200 TFs per image) was calculated. The resulting distribution of distances between TFs was analyzed using custom MATLAB code and fit to a Gaussian curve.

### 4.7. Neonatal Mouse Ventricular Cardiomyocyte Preparation

Neonatal mouse ventricular cardiomyocytes (NMVCMs) were isolated and cultured from littermate P0–P1 mice from the FLNC^f/f^ background using previously described methods [43,44]. NMVCMs were cultured for 48 h at 37 °C and 10% CO_2_ in 35-mm glass-bottom culture dishes with laminin-coated (2 µg/cm^2^) nanopatterned cell culture substrate (CuriBio, Seattle, WA, USA, product number ANFS-0001). Cell culture media (62% Gibco Dulbecco’s Modified Eagle Medium, 20% Medium 199, 10% Horse Serum, 5% Fetal Bovine Serum, 0.8% 100× Penicillin/Streptomycin, 0.8% 100× Glutamine, and 0.8% 1 M HEPES) was replaced every 24 h. At hour 48, NMVCMs were infected with an adenovirus at 200 MOI that expressed either β-galactosidase (ad-LacZ) as a control or Cre-recombinase (ad-Cre) to knock out FLNC in vitro. After 72 h (96 total hours in culture), NMVCMs were fixed and stained for immunofluorescence imaging as described in Section 4.5.

### 4.8. Atomic Force Microscopy

Control and FLNC-knockdown NMVCMs were prepared as described in Section 4.7 and cultured on laminin-coated micropatterned PDMS [43]. Atomic force microscopy (AFM) was performed on an MFP-3D Bio Atomic Force Microscope (Oxford Instruments) mounted in a Ti-U fluorescent inverted scope (Nikon Instruments, Melville, NY) and used Asylum Research 13, Igor Pro 6.34A software. Nanoworld PNP-TR tips were calibrated for their spring constant using the thermal noise method and used for probing the FLNC-knockdown NMVCMs and their controls. A trigger force of 2 nN, an approach Velocity constant of at 2 µm/s, and a force-distance of 6 µm were used to generate a force map with 12 points across 5 µm^2^ [45]. Asylum Research 13, Igor Pro 6.34A software was used to calculate the slope of the deflection-indentation relationship using the Hertz equation (Hertz, H. Ueber den kontakt elastischer koerper. *J. fuer die Reine Angew. Math.* **92**, 156 (1881)) and taken as the Young’s Modulus, or stiffness, (in kPa) of the cell membrane/cortical cytoskeleton. Poor fits to the deflection-indentation were excluded and the average Young’s modulus was calculated from the remaining force map points. One cell was measured per force map.

### 4.9. Spatially Explicit Half-Sarcomere Model

To predict the effects of a compressed myofilament lattice on twitch tension in cardiac muscle (Figure 5) we used a modified version of our previously described [25,26] model to simulate cardiac muscle twitches (Figure S2). A calcium transient was simulated based on experimental measurements of calcium dynamics within the myofilament lattice of adult cardiomyocytes [46]. We varied the face-to-face distance between thick filaments (TFs) from 12–22 nm (as done previously [25]) which corresponds to center-to-center TF-TF distances of 27–36 nm for a TF diameter of 15 nm (our unpublished measurements) and kept the sarcomere length constant at 1.85 µm based on our measurements (Figure 3f). For each TF-TF distance, 50 isometric twitches force-time transients of 550 ms were simulated with a time-step of 0.01 ms and averaged (Figure S2). The peak twitch force was taken as a 10-ms average surrounding the maximum value of the twitch force-time trace for each TF-TF distance.

### 4.10. Spatially Explicit Cell-Level Model

To explore the potential role of the mechanical properties of inter-myofibril Z-disk connectivity in maintaining sarcomere organization, we developed a multibody dynamics model of a single cardiomyocyte using MSC software (ADAMS 2019.2; Newport Beach, CA, USA), which consists of 1000 sarcomeres (50 rows × 20 columns) represented by linear springs with a stiffness coefficient of 35 nN/µm [47] and a damping coefficient of 3.5 nN·s/µm. The initial length for the sarcomeres was sampled from the distribution of our experimental measurements of adult cardiomyocytes (Figure 3f). Subsequent sarcomeres in each row were linked together using rigid links representing Z-disks 1 µm long and 0.1 µm wide [48], connected by a torsional and linear spring (Figure 6b). The solver parameters (GSTIFF integrator) [49] included an integrator error of 0.001 and a simulation step size of 10 ms. Each simulation took ~55 min to complete using a desktop computer (2 × 2.20 GHz Intel Xeon 4114 Processor) with 64 GB of RAM.

The torsional spring stiffness was varied from 0–14 nN·µm/deg (with a constant linear stiffness of 35 nN/µm) and the simulation was run for 1 s of simulation time. The Z-disk dispersion angle was measured relative to the vertical axis, analogous to our experimental measurements. Similarly, we measured the maximum Z-disk bundling by determining the maximum number of Z-disks aligned vertically with a displacement distance ≤0.05 µm (i.e., half the Z-disk width) for linear spring stiffnesses between 0 and 1 nN/µm.

## 5. Conclusions

Our work identifies FLNC as a key regulator of the multiscale ultrastructure of cardiomyocytes, and therefore plays an important role in maintaining systolic mechanotransmission pathways. Namely, we present new evidence suggesting that systolic deficiencies in hearts with a loss of FLNC are mediated by disrupted inter-myofibril connectivity at the Z-disk and reduced transverse cell stiffness, which causes sarcomere misalignment and altered myofilament lattice geometry that, together, reduce longitudinal force development in myocytes. These alterations to multiscale structure-function relationships in the heart may be critical to the pathogenesis of DCM, opening new possible research avenues that investigate potential preventative or restorative therapies. Thus, our work provides new insights into the molecular mechanisms by which dysfunctional FLNC promotes contractile abnormalities in the development of DCM, as well as a new platform for using quantitative multiscale structural analyses of failing cardiac tissue to inform mechanistic computational models that predict the ultrastructure-function relationships of the heart.

## Data Availability

Not applicable.

## Supplementary Materials

The following supporting information can be downloaded at: https://www.mdpi.com/article/10.3390/ijms23020871/s1.
