# Supplementary material for: Subcellular Remodeling in Filamin C Deficient Mouse Hearts Impairs Myocyte Tension Development during Progression of Dilated Cardiomyopathy
Source: Int J Mol Sci. 2022 Jan 14;23(2):871. doi: 10.3390/ijms23020871 (PMC8779483; doi:10.3390/ijms23020871)
Supplement: Supplementary file 1 [file ijms-23-00871-s001.zip › ijms-1514422-supplementary.pdf]

Supplemental Information – Powers et al.

Supplemental Tables

Table S1. Primary antibodies used for immunofluorescence imaging of adult cardiomyocytes.

1° Antibody	Species	Dilution	Vendor	Product Number
anti-FLNC	Rabbit	1:50	Sigma-Aldrich	HPA006135
anti-α-Actinin	Mouse	1:500	Sigma-Aldrich	A7811

Table S2. Secondary antibodies used for immunofluorescence imaging of adult cardiomyocytes.

2° Antibody	Species	Label	Dilution	Vendor	Product Number
anti-Rabbit	Goat	Texas Red	1:500	ThermoFisher	T2767
anti-Mouse	Goat	Alexa Fluor 700	1:500	ThermoFisher	A21036

Supplemental Figures

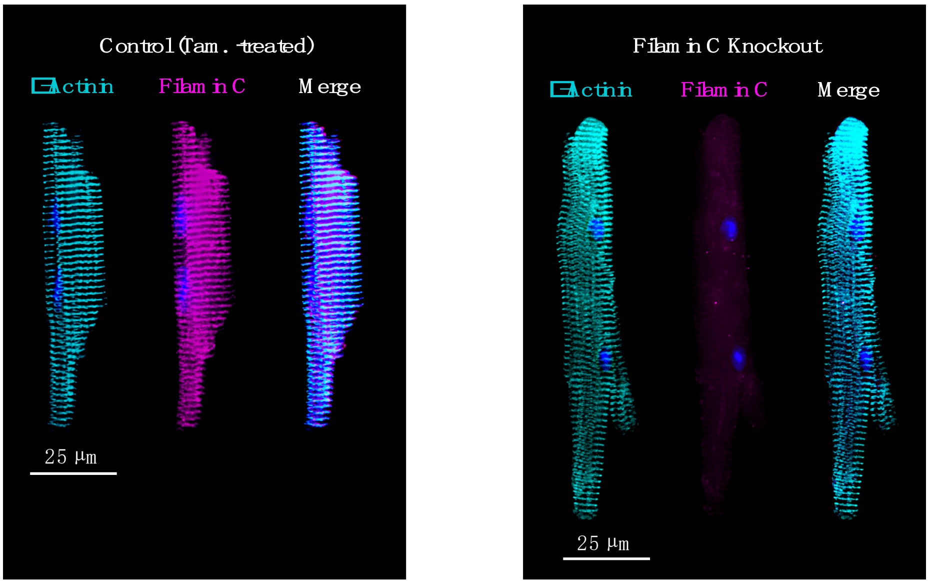

Figure S1. Representative immunofluorescent images of control (left) and FLNC-knockout (right) adult mouse ventricular cardiomyocytes, stained for the Z-disk protein α-actinin (cyan), FLNC (magenta), and nuclei (DAPI, blue). Cardiomyocytes were isolated from the ventricles of 10-week-old adult male and female littermates 2 weeks post-injection with tamoxifen (injections at 8 weeks of age). See Materials and Methods for details.

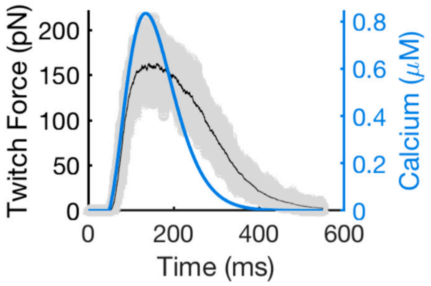

Figure S2. Computational model of cardiac twitch force. The blue trace corresponds to the calcium transient within the myofilament lattice (right abscissa) and the black trace is the average twitch force (left abscissa) over 50 twitch simulations (gray traces).
